# Report on BrainChild hydrocephalus conference

**DOI:** 10.1186/1743-8454-4-4

**Published:** 2007-04-19

**Authors:** Conrad Johanson

**Affiliations:** 1 Department of Clinical Neurosciences, Brown Medical School, Providence. RI 02903, USA

## Abstract

A report of a meeting sponsored by the BrainChild Foundation on the challenges for hydrocephalus researchers to provide the information required for better management of cerebrospinal fluid disorders.

## Background

Investigators from several hydrocephalus research groups met at the Brown Medical School in Providence, Rhode Island to discuss ways to more effectively integrate the biophysical and biochemical phenomena associated with the brain, cerebrospinal fluid (CSF) and cerebrovascular fluids; and the epithelial and endothelial membranes that demarcate these compartments. The two-day meeting on November 14^th ^and 15th was sponsored by the BrainChild Foundation of Carefree, Arizona and hosted by Neurosurgery chief John Duncan and Conrad Johanson in the Department of Neurosurgery at Rhode Island Hospital.

## Discussion

Following the opening presentation on the aging choroid plexus-CSF system by conference organizer C. Johanson, there were talks by Gerald Silverberg, Deborah Grzybowski (Ohio State) and Ed Stopa, on the topics of compromised toxic metabolite clearance in NPH, new preparations for studying the arachnoid membrane, and the destruction of the cerebral vessels in Alzheimer's disease, respectively. The afternoon session began with discussion by Pat McAllister (Wayne State) of glial responses to NPH that can impact the cerebrovascular system and intracranial pulsatility. Per Eide from Norway talked about biophysical/biochemical linkage analysis from simultaneous ICP monitoring and microdialysis. Stony Brook investigators Mike Egnor and Mark Wagshul discussed the notch filter hypothesis (cerebral Windkessel mechanism) and flow MRI exploration of pulsatile intracranial CSF movements. Concluding the presentations were Joe Madsen and E-H. Park from Harvard who updated the group on genes of mechano-biological interest and the analysis of wave amplitude pressures in NPH.

Several 'up-and-coming' CSF/blood-brain barrier researchers also attended the conference: Erin McCormack, John Donahue, Carolyn Black, Laurel Fleming and Kelley Deren.

## Conclusion

BrainChild Foundation president, Curt Stewart, challenged the group to come together more effectively to translate the animal model findings into better hydrocephalus treatments. The meeting culminated with lively and optimistic brainstorming about the desirability of cooperative inter-institutional ventures to analyze complex CSF pressure waves for the purpose of expediting the management of CSF disorders.

## Competing interests

The author(s) declare that they have no competing interests.

## Authors' contributions

Sole author

